# Atmospheric Cold Microwave Argon Plasma for Decontamination of Dental Implant Surfaces: An In Vitro Experimental Study

**DOI:** 10.3390/jfb17050211

**Published:** 2026-05-01

**Authors:** Todor Bogdanov, Nadja Radchenkova, Raya Grozdanova, Dimitar Kosturkov, Todor Uzunov

**Affiliations:** 1 Department of Medical Physics and Biophysics, Faculty of Medicine, Medical University of Sofia, St. Georgi Sofiyski Str. No. 1, 1431 Sofia, Bulgaria; 2 Department of General Microbiology, The Stephan Angeloff Institute of Microbiology, Bulgarian Academy of Sciences, Acad G. Bonchev Str. Bl. 26, 1113 Sofia, Bulgaria; 3 Department of Conservative Dentistry, Faculty of Dental Medicine, Medical University of Sofia, St. Georgi Sofiyski Str. No. 1, 1431 Sofia, Bulgaria; 4 Department of Prosthetic Dental Medicine, Faculty of Dental Medicine, Medical University of Sofia, St. Georgi Sofiyski Str. No. 1, 1431 Sofia, Bulgaria

**Keywords:** microwave plasma, cold atmospheric plasma, dental implants, peri-implantitis, *Streptococcus mutans*, *Chromohalobacter canadensis*, antimicrobial plasma treatment, reactive oxygen and nitrogen species

## Abstract

Dental implants are widely used to replace missing teeth, but peri-implantitis remains a major biological complication associated with bacterial biofilm formation on implant surfaces. The increasing incidence of peri-implant infections underscores the need for alternative antimicrobial strategies that effectively decontaminate complex titanium implant surfaces. This study evaluated the inhibitory effect of low-temperature microwave argon plasma on bacteria in an experimental model simulating peri-implant conditions and compared the responses of microorganisms with different biological characteristics. A 3D-printed mandibular bone segment model with an inserted Straumann BLX Roxolid^®^ dental implant was used to reproduce the peri-implant environment. Bacterial suspensions of *Streptococcus mutans* NBIMCC 1786 and the extremophilic bacterium *Chromohalobacter canadensis* NBIMCC 9077 have been exposed to a microwave non-equilibrium argon plasma jet (2.45 GHz, atmospheric pressure) for 1–7 min. Optical density measurements and colony growth analysis were used to assess antimicrobial effects. Plasma treatment induced a pronounced reduction in bacterial growth during the early post-treatment period. In *C. canadensis*, growth inhibition reached a plateau (~47–55% at 24 h) regardless of exposure time. In contrast, *S. mutans* showed a nonlinear response, with stable inhibition after short exposures (1–3 min) and partial recovery after longer treatments (5–7 min). These findings indicate that microwave argon plasma exhibits significant antimicrobial activity under controlled in vitro conditions, although its effectiveness depends on microorganism-specific biological characteristics. Because the present model was based on simplified single-species systems, direct clinical extrapolation remains limited and should be addressed in future studies using polymicrobial peri-implant biofilm models.

## 1. Introduction

Dental implants are nowadays the gold standard for the reconstruction of partial and complete tooth loss, with high implant survival rates and noticeable improvements in patients’ quality of life [1,2]. Parallel to the exponential increase in implantological treatment in contemporary clinical practice, an increase in the incidence of biological complications is observed, among which peri-implant diseases occupy a leading position [3,4].

Clinically, peri-implantitis presents as a plaque-driven inflammatory process affecting the tissues surrounding a functional dental implant. Identifying localized mucosal inflammation and the progressive loss of the supporting alveolar bone remain the clinical cornerstones for diagnosing this condition [5,6,7]. However, when examining the current literature, one finds that prevalence estimates for this pathology vary widely; indeed, some epidemiological surveys and systematic reviews report rates of 40–45% [8,9,10]. This lack of consistency in the data may be due to the use of non-standardized diagnostic thresholds across studies. Nevertheless, even accounting for these differences, pessimistic estimates indicate that peri-implantitis remains a persistent and widely distributed complication throughout the clinical life of dental restorations, determining the significant clinical and socio-economic impact of the disease and highlighting the need to develop more effective methods for controlling infection around implant surfaces.

The pathogenesis of peri-implantitis is closely associated with the formation and evolution of bacterial biofilms on the implant surface, as well as with the macro- and microtopographic characteristics of titanium implants [11,12]. The oral microbiome is a complex, dynamic ecosystem comprising more than 600 microbial species that interact within multispecies biofilms [13]. Among them, streptococci, lactobacilli, and other opportunistic microorganisms play a key role in the development of both dental caries and periodontal-like diseases [14,15,16].

*Streptococcus mutans* is a facultatively anaerobic Gram-positive coccus recognized as one of the main etiological factors in dental caries [17,18]. Crucial to the architecture of dental plaque, *S. mutans* establishes itself within the human oral cavity by colonizing hard tooth surfaces. Its profound cariogenic potential arises from a specialized metabolic capacity to convert sucrose into extracellular glucan polymers. Rather than simply aiding initial attachment to the substrate, these polymers actively drive the in situ formation of a resilient extracellular matrix. This structural framework is fundamental for the maturation of the entire biofilm. In addition, this microorganism exhibits pronounced acidogenicity—the ability to metabolize a wide range of carbohydrates into organic acids—as well as aciduricity, which enables survival under low-pH conditions [19].

Although *S. mutans* is not the only microorganism involved in the etiopathogenesis of dental caries, numerous studies indicate that it can modify the local microbial environment by forming an EPS-rich, low-pH ecosystem that favors the development of other acidogenic and aciduric bacterial species. As a human pathogen, *S. mutans* is also associated with subacute bacterial endocarditis, a life-threatening inflammatory disease of the heart valves. Certain strains have also been linked to extraoral pathologies, including cerebral microhemorrhages, IgA nephropathy, and atherosclerosis [20].

Strains of *Streptococcus mutans* are classified into four serological groups (c, e, f, and k) based on the composition of the cell-surface rhamnose–glucose polysaccharide. Approximately 75% of strains isolated from dental plaque belong to serotype c, about 20% to serotype e, and the remaining 5% are distributed between serotypes f and k [21].

An important characteristic of *S. mutans* is its ability to adapt to sudden and significant changes in dental plaque conditions by developing the so-called acid tolerance response. This complex transcriptional and physiological mechanism includes cytoplasmic buffering, modifications in membrane fatty acid composition, and protective mechanisms of the cellular apparatus against acid-induced damage [19,22,23]. This high adaptability poses a serious challenge for modern medicine and dentistry, which have traditionally addressed it through chemical antimicrobial agents.

Current therapeutic strategies for peri-implantitis include mechanical and chemical decontamination, as well as various nonsurgical and surgical protocols aimed at controlling infection and achieving re-osseointegration [24,25]. Despite the availability of numerous treatment techniques, including implantoplasty and various chemotherapeutic agents, the complete elimination of bacterial biofilms and the stable restoration of the supporting bone remain significant challenges, particularly in implants with modified and moderately rough surfaces [11,12,26].

In recent years, low-temperature (cold) plasma has attracted considerable interest as an innovative physical technology with potential applications in medicine and dentistry. Particular attention has been given to cold atmospheric plasma and microwave argon plasma, which have demonstrated the ability to effectively disrupt oral bacterial biofilms on titanium surfaces, including those formed by mixed microbial communities [27,28,29,30,31,32].

In addition to its direct antimicrobial action, evaluating potential cellular effects associated with oxidative and nitrosative stress is of significant importance. In this context, the formation of 3-nitrotyrosine is considered an important marker of protein modification and a potential indicator of apoptosis induction [33,34,35].

The microorganisms included in the present study were selected to examine cellular responses to plasma exposure in bacteria with diverse biological characteristics. The opportunistic oral pathogen *Streptococcus mutans* NBIMCC 1786 was used as a representative species of the oral microbiota. In parallel, the moderately halophilic extremophile *Chromohalobacter canadensis* NBIMCC 9077 was included as a comparative model organism adapted to survive in highly saline environments.

*Chromohalobacter canadensis* is a Gram-negative, non-spore-forming aerobic bacterium that grows optimally at sodium chloride concentrations of approximately 8–10%. Its growth has been reported over a temperature range of 5–45 °C and within a pH range of 5.0–10.0. The organism is chemoorganotrophic and is typically found in hypersaline environments such as the Dead Sea and marine saltworks [36]. In the present work, the response of this extremophilic bacterium to plasma exposure was analyzed and compared with that observed for *Streptococcus mutans*.

Although interest in low-temperature plasma technologies for antimicrobial applications in dentistry has increased substantially in recent years, experimental evidence regarding the effectiveness of microwave argon plasma in peri-implant models remains limited. In addition, the responses of microorganisms with different cellular organization and physiological adaptations to plasma treatment, particularly with regard to dose–time relationships and post-exposure recovery dynamics, have not yet been fully elucidated. Reactive oxygen and nitrogen species (RONS) generated by plasma are known to possess antimicrobial potential and may induce oxidative and nitrosative damage to cellular components. Because peri-implant diseases are associated with complex polymicrobial biofilms, simplified in vitro systems cannot reproduce the full ecological and clinical complexity of the oral environment. However, controlled single-species models remain useful as an initial experimental step for identifying microorganism-specific response patterns under standardized conditions. On this basis, the present study aimed to evaluate the bacterioinhibitory effect of low-temperature microwave argon plasma in a simplified experimental model simulating peri-implant conditions and to compare the responses of two microorganisms with distinct biological characteristics: *Streptococcus mutans* NBIMCC 1786 and the extremophilic bacterium *Chromohalobacter canadensis* NBIMCC 9077.

## 2. Materials and Methods

### 2.1. Experimental Design and Implant Model System

**Figure 1:**
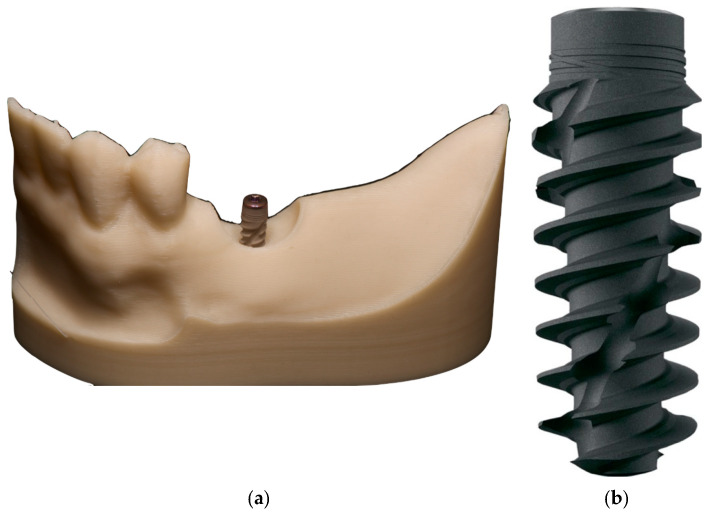
3D-printed model of a mandibular bone segment with a dental implant (**a**) and dental implant Straumann BLX RB, Roxolid^®^ (Straumann USA LLC, Andover, MA, USA) with SLActive^®^ surface, 16 mm/4 mm (**b**).

A model system consisting of a dental implant with a cover screw, placed in a specially designed 3D-printed mandibular bone segment model, was developed. To simulate a clinical situation in the oral cavity, a socket-type jaw simulation model with an inserted dental implant was used (Figure 1a).

A Straumann BLX RB dental implant (16 mm/4 mm) was used in the experiments (Figure 1b). The implant is manufactured from the implant material Roxolid^®^ with an SLActive^®^ surface. The implant design includes a fully conical body with self-cutting threads and a channel to redistribute bone chips, as well as microthreads at the implant neck to reduce stress on the cortical bone.

Roxolid^®^ is a patented titanium–zirconium alloy representing a binary metal system that combines the biocompatibility of titanium with the increased mechanical strength of zirconium in a composition of 85% titanium (Ti) and 15% zirconium (Zr).

The SLActive^®^ surface is characterized by sandblasted and acid-etched microtopography, high surface energy, hydrophilicity, and accelerated adsorption of proteins and fibrin.

### 2.2. Microorganisms and Culture Conditions

#### 2.2.1. Bacterial Strains

The bacterial strains *Streptococcus mutans* NBIMCC 1786 (ATCC 25175), serological group C, and *Chromohalobacter canadensis* NBIMCC 9077 (ATCC 43984), strain HX, were obtained from the National Bank for Industrial Microorganisms and Cell Cultures (NBIMCC), Sofia, Bulgaria, and were used as test microorganisms in the present study.

Stock cultures were stored at −20 °C by adding 500 μL of sterile 100% glycerol to 500 μL of bacterial culture in a UniINCU 20 cool incubator (LIG Systems) (Lab Logistics Group GmbH, Meckenheim, Germany).

#### 2.2.2. Culture Media

The culture media used contained (%):

- *Streptococcus mutans*: Brain Heart Infusion Broth (BHIB, Sigma-Aldrich, Ankara, Turkey)—3.7; pH = 7.4
- *Chromohalobacter canadensis*: yeast extract (Fisher Scientific, Waltham, MA, USA)—0.5; NaCl—10; lactose (Sigma-Aldrich)—1.0; tryptone—1.0; pH = 7.2

For solid media, agar (3%) was added.

#### 2.2.3. Cultivation Conditions

Erlenmeyer flasks containing 100/25 mL of sterile liquid culture medium were inoculated with 5% (*v*/*v*) bacterial culture and incubated under the following conditions:

- *Streptococcus mutans*: 37 °C, 150 rpm, 48 h
- *Chromohalobacter canadensis*: 30 °C, 150 rpm, 48 h

Incubation was performed using a shaking water bath (Nuve ST 30) (NUVE, Saracalar Mahallesi Saracalar Kümeevleri No:4/2 Akyurt 06750 Ankara, Turkey).

All experiments were conducted using a fresh 24 h inoculum.

Optical density (OD660) of the samples was measured immediately after cultivation, before treatment, and at 24 h and 96 h after treatment, using sterile culture medium as a control. Measurements were performed with a UV-52-SCAN spectrophotometer (ONDA). (Giorgio Bormac S.r.l. Via della Meccanica, Carpi, Italy).

Under aseptic conditions, 24 h after treatment, samples from all bacterial suspensions were plated on solid culture media. Petri dishes were incubated at the corresponding temperatures and observed for 72 h. The results from spectrophotometric measurements and colony formation on solid media were recorded, compared, and analyzed.

### 2.3. Inoculation of the Implant Model System

**Figure 2:**
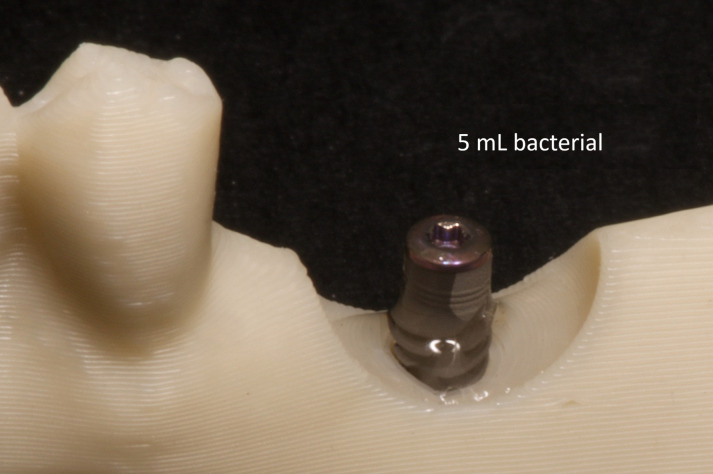
Inoculation of the implant surface with 5 mL of bacterial suspension.

To simulate a clinical situation in the oral cavity, a socket-type jaw simulation model with a dental implant was used. A 5 mL volume of bacterial suspension was added to the cavity around the implant (Figure 2), ensuring immersion of the implant surface and recreating a peri-implant environment.

### 2.4. Plasma Treatment Setup

**Figure 3:**
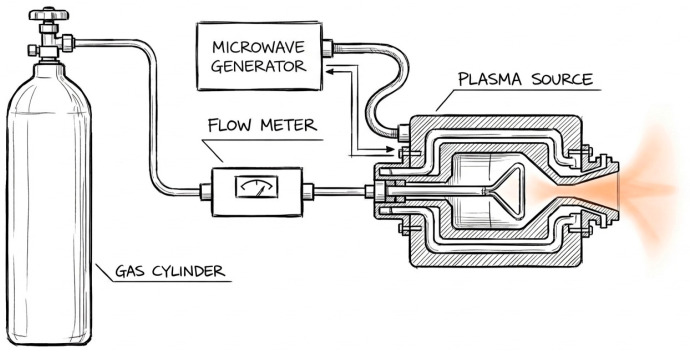
Microwave non-equilibrium plasma jet source operating at a frequency of 2.45 GHz and atmospheric pressure.

The infected model system was treated with cold microwave argon plasma to determine optimal treatment parameters and investigate time-dependent effects. The experimental studies were carried out using a microwave non-equilibrium plasma jet source, operating at 2.45 GHz and at atmospheric pressure (Figure 3).

The device consists of a solid-state microwave generator connected to a coaxial plasma module, with a metal antenna positioned axially. Argon with a purity of 99.996% was used as the working gas and supplied to the plasma module via a mass flow meter and flow regulator.

In the present experiments, the argon flow rate was fixed at 5 L/min, and the microwave power supplied to the source was 10 W. These parameters were selected from a range previously characterized as safe and suitable for biomedical applications, yielding low gas temperatures and a stable plasma jet.

The plasma source was mounted on a laboratory stand and positioned perpendicular to the liquid surface above the implant. The tip of the visible plasma jet was maintained in the discharge afterglow region at a small distance above the suspension surface (1–2 mm), without direct electrical contact with the model.

**Figure 4:**
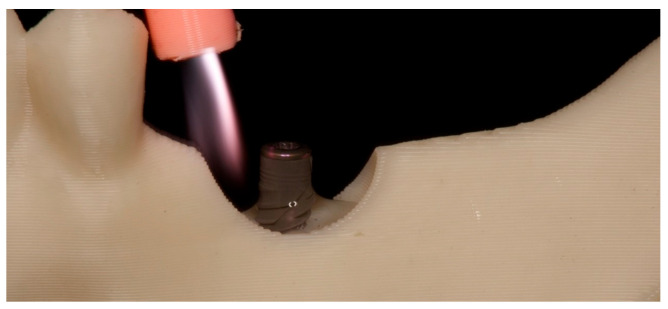
Treatment of the surface of the bacterial suspension with a flow of argon non-equilibrium plasma.

Under these conditions, treatment was performed using a non-equilibrium argon plasma containing reactive oxygen and nitrogen species (RONS), which surrounded the implant and reached the liquid surface (Figure 4).

The experimental setup combined a realistic geometry (socket-type jaw model with an implant) with controlled plasma parameters, including a fixed gas flow rate, a defined microwave power, and a stable plasma jet length. A combination of discharge conditions enabled reproducible treatment of 5 mL of bacterial samples surrounding the implant and quantitative comparison of the antibacterial effect of plasma exposure.

### 2.5. Quantitative Analysis of Bacterial Growth

Quantitative changes in bacterial growth under different plasma treatment conditions were evaluated using both spectrophotometric and culture-based methods. Optical density (OD) of the samples was measured at 600 nm and 660 nm using a UV-52-SCAN spectrophotometer (ONDA), with sterile culture medium used as a blank control.

In addition to absolute OD values, the inhibitory effect of plasma treatment was assessed by calculating the percentage inhibition relative to the corresponding untreated control samples. For each experimental condition, inhibition was determined using a standard relative reduction approach (Equation (1)).


(1) $${\%}_{\mathrm{inhibition}}=100\cdot (1-\frac{{OD}_{\mathrm{treated}}}{{OD}_{\mathrm{control}}})$$

The calculated inhibition values were used to compare the antimicrobial effectiveness of different plasma exposure times. The results from spectrophotometric measurements were further supported by a qualitative assessment of colony formation on solid culture media, enabling a combined evaluation of bacterial growth dynamics and viability.

### 2.6. Statistical Analysis

All experimental measurements were performed in independent experimental series corresponding to the different plasma exposure times. Each series consisted of five parallel replicates (*n* = 5) under identical experimental conditions. Optical density (OD600 and OD660) values obtained by spectrophotometry, together with colony growth data from solid culture media, were recorded and used to evaluate bacterial growth following plasma treatment.

Data are presented as mean ± standard deviation (SD). Statistical differences between experimental groups corresponding to different plasma treatment times were assessed using one-way analysis of variance (ANOVA). Differences were considered statistically significant at *p* < 0.05.

The analysis was performed by comparing optical density values measured immediately after cultivation and at defined time points after treatment (3 h, 24 h, and 96 h, depending on the experimental series). Variations in bacterial growth were evaluated both quantitatively, using OD measurements, and qualitatively, by comparing colony formation on solid media.

GenAI (https://gpai.app/visualizer—AI STEM visualizer) (accessed on 17 March 2026) was used for preparing Figure 3.

## 3. Results 

### 3.1. Analysis of Optical Density Measurements After Microwave Plasma Treatment

Although optical density measurements were performed at multiple wavelengths (500–700 nm), the analysis presented here focuses on OD600, which is a standard parameter for estimating bacterial growth in liquid cultures [37]. Measurements at 660 nm were also performed and are included in the summary tables for comparative purposes (Table 1, Table 2, Table 3 and Table 4).

### 3.2. Analysis of Experimental Data

#### 3.2.1. *Chromohalobacter canadensis*

**Figure 5:**
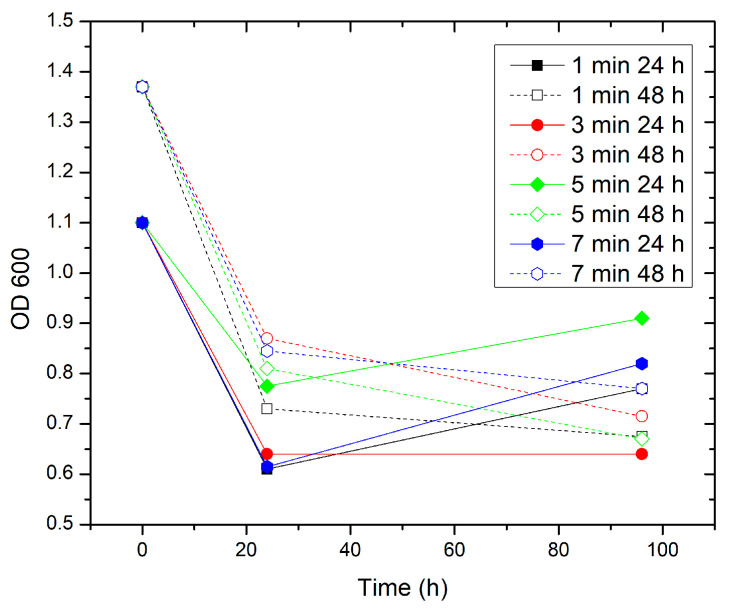
Comparison of OD600 for different treatment times, averaged by subgroups for *Chromohalobacter canadensis study*.

The graphical comparison of OD600 dynamics for *Chromohalobacter canadensis* (Figure 5) across different plasma treatment times reveals a consistent pattern for both cultures treated after 24 h and after 48 h of growth. Regardless of the initial optical density, which is higher for the 48 h cultures, all experimental conditions show a sharp decrease in OD600 during the first 24 h after exposure, followed by partial recovery or stabilization between 24 and 96 h (Figure 5). This pattern indicates that the most pronounced reduction in bacterial growth occurs during the early post-treatment period. The detailed results for each experimental group are provided in Appendix A.

For the individual treatment durations (1, 3, 5, and 7 min), small quantitative differences are observed; however, they do not alter the overall dynamics of the response. In the 24 h input culture, treatment for 1 min yields the largest initial decrease in OD600, whereas in the 48 h input culture, the most pronounced decrease occurs after 7 min of treatment (Figure 5). Despite these variations, the curves for the two input cultures remain almost parallel over time.

After 24 h, the differences among the individual treatment times begin to level out. In some groups (e.g., 1 and 7 min), a slight increase in OD600 is observed between 24 and 96 h, whereas in others, especially 3 and 5 min for the 48 h input culture, OD600 values show slight stabilization or a further decrease (Figure 5).

When comparing the four treatment times, each represented by two averaged curves (for 24 h and 48 h input cultures), the curves show similar overall shapes and trends across all experimental conditions (Figure 5).

For the 1 min treatment, the curves for both input cultures start at different initial values but decrease sharply to approximately 0.6–0.7 at 24 h. After 24 h, a slight recovery is observed, with a more pronounced effect in the 24 h input culture. For the 3 min treatment, the decrease in OD600 at 24 h is somewhat smaller than that observed after 1 min treatment. At 96 h, the 24 h input culture shows slight stabilization, whereas the 48 h input culture continues to decline. For the 5 min treatment, the curves remain relatively stable between 24 and 96 h after the initial decrease. For the 7 min treatment, the largest decrease at 24 h is observed in the 48 h input culture, followed by a relatively stable pattern up to 96 h (Figure 5).

Overall, treatment times follow a common pattern: a sharp decrease in OD600 during the first 24 h, followed by moderate recovery or stabilization through 96 h (Figure 5).

In the present context, “dose–time dependence” refers to the effect of the “plasma dose” (at constant power) multiplied by the exposure time on the bacterial population, as evaluated by OD600. Since the power and geometry of the plasma source are fixed, the treatment time effectively serves as the “dose”.

When examining the averaged OD600 values at 24 h for the 1-, 3-, 5-, and 7 min treatments (combined k24 and k48), they are approximately 0.67, 0.76, 0.79, and 0.73, respectively, compared with the control (k24 ≈ 1.50). These values correspond to approximately 55% inhibition at 1 min, ~50% at 3 min, ~47% at 5 min, and ~51% at 7 min.

In addition to the absolute OD600 values, relative inhibition compared with untreated controls was also analyzed. At the 24 h time point, all treatment durations (1–7 min) produced strong growth suppression, with average inhibition ranging from approximately 53% to 60% relative to the corresponding controls. One-way ANOVA did not reveal statistically significant differences between the individual exposure times (F = 1.02, *p* = 0.42), indicating the absence of a clear dose–time dependence within this interval. At 96 h, the inhibitory effect was partially reduced, and substantial variability was observed across all treatment durations. Analysis of inhibition at 96 h again showed no significant differences between the groups (F = 0.24, *p* = 0.86). Summary data are presented in Table 1 and Table 2.

To integrate the effect over time, the area under the OD–time curve (AUC) was calculated for the 0–96 h interval. The average AUC values across different treatment times differed by no more than 10–15%. They did not follow a monotonic sequence, confirming that the overall cumulative growth suppression was similar for all tested exposures.

For both wavelengths and at both time points (24 and 96 h), one-way ANOVA did not reveal statistically significant differences between the groups treated for 1, 3, 5, and 7 min (Table 2). Systematized inhibition data for OD600 and OD660 are presented in Table 1.

#### 3.2.2. *Streptococcus mutans*

**Figure 6:**
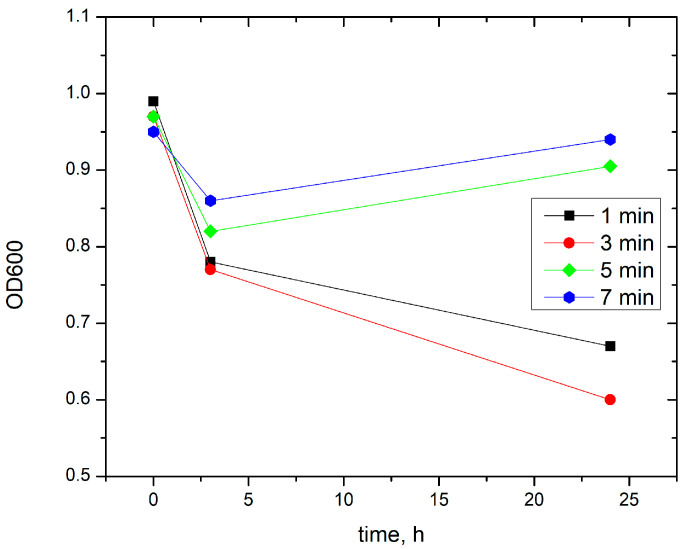
Comparison of OD600 for different treatment times, averaged by subgroups for *Streptococcus mutans study*.

The graphical representation of the results for *Streptococcus mutans* (Figure 6) shows a rapid response of the bacterial culture to plasma treatment within the first 3 h after exposure. Regardless of treatment duration (1, 3, 5, or 7 min), all groups exhibit a sharp decrease in OD600 relative to the initial time point (Figure 6). The detailed results for each experimental group are provided in Appendix B.

At the third hour, the differences among the individual treatment times are relatively small. The lowest values are observed in the groups treated for 3 and 1 min, whereas the 5 and 7 min treatments retain slightly higher optical density values (Figure 6).

Between the third and the twenty-fourth hour, dynamics vary depending on treatment duration. The groups treated for 1 and 3 min continue to show a decrease in OD600, whereas the groups treated for 5 and 7 min show a visible increase in OD600 between 3 and 24 h (Figure 6).

Results of the inhibition and ANOVA analyses are presented in Table 3 and Table 4. At the third hour after exposure, both OD600 and OD660 show a moderate reduction in optical density, while differences between treatment times remain limited. At the twenty-fourth hour, short plasma treatments (1–3 min) maintain moderate inhibition, whereas the longer treatments (5–7 min) show markedly increased variability, including samples with substantial recovery of optical density relative to the control cultures (Table 3).

For OD600, ANOVA yielded statistically significant values at 3 h and 24 h due to zero within-group variability in some measurements (Table 4). For OD660, no statistically significant differences were observed between treatment groups either at 3 h (F = 1.49, *p* = 0.345) or at 24 h (F = 1.32, *p* = 0.381) (Table 4).

The analysis of optical densities at 600 and 660 nm shows a generally consistent pattern in the response of *Streptococcus mutans* to plasma treatment. At the 24 h time point, both measurement wavelengths distinguish between short (1–3 min) and longer (5–7 min) plasma treatments, with increased variability observed in the longer exposure groups (Table 3 and Table 4).

## 4. Discussion

### 4.1. Main Findings

The present study demonstrates that microwave argon plasma induces a pronounced inhibitory effect on bacterial growth during the early post-treatment period in both investigated microorganisms under the controlled conditions of this in vitro peri-implant model. At the same time, the two species exhibited different response patterns under identical plasma parameters, indicating that antimicrobial efficacy is strongly influenced by microorganism-specific biological characteristics. These findings should be interpreted within the boundaries of the experimental design, which was intentionally simplified and was not intended to reproduce the full polymicrobial complexity of peri-implant biofilms in vivo.

### 4.2. Chromohalobacter canadensis

The observed antimicrobial efficacy against *Chromohalobacter canadensis* is notable given the complex surface topography of the Straumann BLX Roxolid^®^ implant used in this model. The SLActive^®^ surface is characterized by sandblasted and acid-etched micro-roughness [11,12], which, while promoting rapid osseointegration, also provides numerous micro-niches that may hinder conventional decontamination procedures. Under these conditions, the plasma treatment produced substantial growth inhibition, with values of approximately 47–55% at 24 h depending on treatment duration (Figure 5; Table 1).

Although all treatment durations (1–7 min) yielded a similar overall pattern of inhibition, differences among individual exposure times were observed primarily in the magnitude of the initial OD600 decrease and in the subsequent recovery dynamics. Short exposures (1 min) were associated with a pronounced initial reduction followed by partial recovery, whereas intermediate exposure times (3–5 min) showed more stable inhibition profiles between 24 h and 96 h. Longer exposure (7 min) resulted in a stronger initial decrease in the 48 h input cultures, followed by relatively stable OD values over time (Figure 5).

The absence of a clear linear dose–response relationship, together with the rapid attainment of an inhibitory plateau, suggests that the antimicrobial mechanism reaches near-maximal efficiency early in the treatment cycle. Even short plasma exposures appear sufficient to achieve strong initial suppression of growth. This observation is consistent with previous reports demonstrating effective biofilm disruption of titanium-associated microbial communities by cold atmospheric plasma [30].

From a dose–time perspective, the data indicate that under the present experimental conditions, the plasma system operates in a region of effect saturation. Extending the exposure time from 1 to 7 min did not yield statistically significant increases in inhibition (Table 2), although differences in recovery dynamics were observed up to 96 h. These findings indicate that treatment duration may influence the persistence of the inhibitory effect rather than its initial magnitude.

### 4.3. Streptococcus mutans

In contrast, *Streptococcus mutans* exhibited a nonlinear response to plasma treatment. Short exposures (1–3 min) were associated with more stable inhibition up to 24 h, whereas longer exposures (5–7 min) showed greater variability and partial recovery of the bacterial population (Figure 6; Table 3 and Table 4). The differences between treatment durations were particularly evident in the post-treatment dynamics. While short plasma exposures resulted in a sustained decrease or stabilization of OD600 values between 3 h and 24 h, longer exposures led to an increase in OD600 during the same interval, indicating activation of recovery processes. This observation suggests that prolonged plasma treatment does not necessarily enhance antimicrobial efficacy and may instead alter the balance between cellular damage and subsequent regrowth.

This pattern may be related to the well-documented acid tolerance and stress response mechanisms of S. mutans, as described by Lemos and Burne [19] and Baker et al. [22]. Prolonged plasma exposure may trigger transcriptional and physiological defense responses, including activation of molecular repair systems and metabolic adaptation pathways, which support post-treatment recovery. The thick peptidoglycan layer of this Gram-positive microorganism and its capacity to modify membrane fatty acid composition [23] may contribute to temporary protection against oxidative stress. As a result, a subpopulation of cells may survive the initial plasma-induced damage and subsequently resume growth, particularly after prolonged exposure.

These findings indicate that, in contrast to *Chromohalobacter canadensis*, the antimicrobial effect of plasma on *Streptococcus mutans* is strongly influenced by treatment duration and is characterized by a balance between inhibitory and adaptive responses.

### 4.4. Comparative Interpretation

The comparative analysis of *Chromohalobacter canadensis* NBIMCC 9077 and *Streptococcus mutans* revealed species-specific differences in response to microwave plasma exposure. These distinctions may be related to the structural and physiological organization of Gram-negative and Gram-positive cell envelopes, as well as to differences in stress adaptation mechanisms. In *C. canadensis*, the inhibitory effect rapidly reached a relatively stable plateau, whereas *S. mutans* demonstrated a more dynamic pattern characterized by early inhibition followed by partial recovery in some treatment groups.

Importantly, these observations should be interpreted as mechanistic findings derived from a simplified single-species experimental system. Peri-implant diseases are driven by complex multispecies biofilms, in which microbial interactions, extracellular matrix formation, nutrient gradients, and host-related environmental factors substantially influence antimicrobial susceptibility. Therefore, the present results do not aim to replicate the full ecology of peri-implantitis. Rather, they provide a controlled first-step analysis of how different microorganisms may respond to microwave argon plasma when examined individually under standardized peri-implant-like conditions.

These findings are consistent with the observations of Bernhardt et al. [32], who noted that the efficacy of cold plasma is strongly dependent on the target microorganism’s cell wall composition and its ability to manage oxidative stress. In this sense, the present study provides a foundation for future investigations in polymicrobial and more clinically representative biofilm systems.

### 4.5. Implications for Plasma Treatment

Taken together, the results indicate that short plasma exposures may be sufficient to achieve substantial antimicrobial effects under the investigated in vitro conditions. Prolonged treatment did not necessarily enhance inhibition and, in the case of *S. mutans*, may even have favored partial recovery. Therefore, the optimization of plasma treatment parameters should take into account microorganism-specific responses rather than relying on a uniform exposure protocol. At the same time, these observations should be validated in polymicrobial biofilm systems before broader conclusions are drawn regarding potential peri-implant applications.

### 4.6. Limitations of the Study

The present study has several limitations. First, the experimental design used simplified single-species bacterial systems and therefore does not reproduce the biological complexity of polymicrobial peri-implant biofilms, which involve interspecies interactions, extracellular matrix development, and ecological cooperation or competition. Second, although the 3D-printed implant model reproduced certain geometric aspects of the peri-implant environment, it did not include saliva, acquired pellicle formation, soft-tissue components, host immune factors, or dynamic oral conditions that may influence microbial behavior and treatment response. Third, the plasma composition and the concentration of generated reactive species were not directly characterized in the present study. Fourth, the microbiological readout was based primarily on optical density measurements and colony growth analysis and did not include molecular or structural biofilm characterization. Finally, the findings are limited to a controlled in vitro proof-of-concept setting and should be interpreted cautiously with respect to clinical translatability.

### 4.7. Future Directions

Future studies should evaluate microwave argon plasma in polymicrobial peri-implant biofilm models involving clinically relevant oral pathogens and more mature biofilm architecture. Additional work should incorporate oral environmental factors such as saliva-derived conditioning films, soft-tissue simulation, and dynamic exposure conditions in order to improve biological realism. Further characterization of plasma-generated reactive species and their relationship to antimicrobial efficacy would also strengthen mechanistic interpretation. Such studies will be essential for determining whether the microorganism-specific effects observed here remain valid under more clinically representative conditions.

## 5. Conclusions

Microwave non-equilibrium argon plasma demonstrated a pronounced antibacterial effect against both investigated microorganisms during the early post-treatment period in this controlled in vitro peri-implant model. In *Chromohalobacter canadensis* NBIMCC 9077, growth inhibition reached a plateau (approximately 47–55% at 24 h) regardless of exposure time, indicating that even short treatments were sufficient to achieve a substantial initial antimicrobial effect under the applied conditions.

In contrast, *Streptococcus mutans* exhibited a nonlinear response, with more stable inhibition after short exposures (1–3 min) and increased variability, including partial recovery, after longer exposures (5–7 min). These findings indicate that the antimicrobial effect of microwave argon plasma is species-dependent and that prolonged exposure does not necessarily enhance inhibition.

The consistency between OD600 and OD660 measurements supports the reliability of the applied turbidimetric approach. However, because the present work was conducted in a simplified single-species in vitro model, the results should be interpreted as proof-of-concept findings rather than as direct evidence of clinical efficacy in peri-implantitis. Future studies should validate these observations in polymicrobial biofilm systems and more clinically relevant peri-implant models.

## Figures and Tables

**Table 1:** Average inhibition (%) during treatment.

Treatment Time, min	OD600—Inhibition Duration 24 h (Mean ± SD)	OD600—Inhibition Duration 96 h (Mean ± SD)	OD660—Inhibition Duration 24 h (Mean ± SD)	OD660—Inhibition Duration 96 h (Mean ± SD)
1	60.5 ± 5.6%	−11.0 ± 40.2%	62.4 ± 6.6%	−23.2 ± 48.9%
3	55.8 ± 5.9%	−1.3 ± 33.1%	57.9 ± 5.8%	−7.1 ± 41.6%
5	52.9 ± 5.6%	−23.7 ± 53.8%	54.7 ± 8.5%	−37.9 ± 54.3%
7	57.3 ± 7.8%	−21.2 ± 35.8%	59.0 ± 8.5%	−27.6 ± 44.4%

**Table 2:** ANOVA analysis between the 1–7 min treatment groups.

Group	ANOVA F	*p*-Value
OD600—24 h	F = 1.02	*p* = 0.42
OD600—96 h	F = 0.24	*p* = 0.86
OD660—24 h	F = 0.736	*p* = 0.55
OD660—96 h	F = 0.289	*p* = 0.83

**Table 3:** Average inhibition (in %) from the treatment time.

Treatment Time, min	OD600—Inhibition Duration 3 h (Mean ± SD)	OD600—Inhibition Duration 24 h (Mean ± SD)	OD660—Inhibition Duration 3 h (Mean ± SD)	OD660—Inhibition Duration 24 h (Mean ± SD)
1	22.0 ± 0.0%	22.7 ± 0.0%	28.7 ± 1.50%	32.2 ± 1.00%
3	23.0 ± 0.0%	23.9 ± 0.0%	36.2 ± 0.0%	38.5 ± 0.0%
5	18.0 ± 0.0%	19.6 ± 0.0%	3.7 ± 14.29%	6.8 ± 10.77%
7	14.0 ± 0.0%	15.2 ± 0.0%	0.0 ± 39.12%	−0.8 ± 32.60%

**Table 4:** ANOVA results about OD600 and OD660.

Group	ANOVA F	*p*-Value
OD600—3 h	not defined due to zero within-group variance	<0.0001
OD600—24 h	not defined due to zero within-group variance	<0.0001
OD660—3 h	1.49	0.345
OD660—24 h	1.32	0.381

## Data Availability

The original contributions presented in the study are included in the article, further inquiries can be directed to the corresponding author.

## Abbreviation

The following abbreviation is used in this manuscript:

OD	optical density

## Appendix A

The Appendix contains the experimental optical density data for the samples containing *Chromohalobacter canadensis*.

**Table A1:** Probe index of the the samples containing *Chromohalobacter canadensis*.

Probe Number	Decriptor
k1	culture medium
k24	Input 24 h incubation
k48	Input 48 h incubation

The indices (e.g., 1.1–7.4) correspond to individual samples within each experimental series. For *Chromohalobacter canadensis*, four experimental series (1, 3, 5, and 7 min) are presented, each consisting of five independent replicates (n = 5). For *Streptococcus mutans*, two experimental series are shown following the same experimental design. The values presented in the tables correspond to representative or averaged results derived from these replicates for clarity. For example, indices 1.1–1.4 represent summarized data for the experimental group treated for 1 min, while 7.1–7.4 correspond to the group treated for 7 min.

**Table A2:** Probe discharge conditions for the samples containing *Chromohalobacter canadensis*.

Probe Index				Treatment Time, s	MW Power, W	Ar Gas Flow, L/min
k24		k48		0	0	0
1.1	1.2	1.3	1.4	60	10	5
3.1	3.2	3.3	3.4	180	10	5
5.1	5.2	5.3	5.4	300	10	5
7.1	7.2	7.3	7.4	420	10	5

**Table A3:** OD per sample in 0 h interval for the samples containing *Chromohalobacter canadensis*.

λ, nm	k1	k24	k48	1.1	1.2	1.3	1.4	3.1	3.2	3.3	3.4	5.1	5.2	5.3	5.4	7.1	7.2	7.3	7.4
500	0	1.35	1.5	1.35	1.35	1.5	1.5	1.35	1.35	1.5	1.5	1.35	1.35	1.5	1.5	1.35	1.35	1.5	1.5
520	0	1.11	1.4	1.11	1.11	1.4	1.4	1.11	1.11	1.4	1.4	1.11	1.11	1.4	1.4	1.11	1.11	1.4	1.4
600	0	1.1	1.37	1.1	1.1	1.37	1.37	1.1	1.1	1.37	1.37	1.1	1.1	1.37	1.37	1.1	1.1	1.37	1.37
620	0	1.1	1.34	1.1	1.1	1.34	1.34	1.1	1.1	1.34	1.34	1.1	1.1	1.34	1.34	1.1	1.1	1.34	1.34
660	0	0.96	1.23	0.96	0.96	1.23	1.23	0.96	0.96	1.23	1.23	0.96	0.96	1.23	1.23	0.96	0.96	1.23	1.23
670	0	0.92	1.23	0.92	0.92	1.23	1.23	0.92	0.92	1.23	1.23	0.92	0.92	1.23	1.23	0.92	0.92	1.23	1.23
700	0	0.9	1.22	0.9	0.9	1.22	1.22	0.9	0.9	1.22	1.22	0.9	0.9	1.22	1.22	0.9	0.9	1.22	1.22

**Table A4:** OD per sample in a 24 h interval for the samples containing *Chromohalobacter canadensis*.

λ, nm	k1	k24	k48	1.1	1.2	1.3	1.4	3.1	3.2	3.3	3.4	5.1	5.2	5.3	5.4	7.1	7.2	7.3	7.4
500	0	1.64	1.96	0.8	0.63	1	0.83	0.75	0.75	0.94	1	0.88	1	0.9	1	0.85	0.68	0.9	1.2
520	0	1.62	1.96	0.78	0.62	0.9	0.79	0.73	0.73	0.9	1.1	0.85	0.98	0.95	0.98	0.8	0.66	0.8	1.2
600	0	1.5	1.9	0.68	0.54	0.82	0.64	0.64	0.64	0.74	1	0.74	0.81	0.8	0.82	0.68	0.55	0.69	1
620	0	1.45	1.86	0.66	0.52	0.8	0.62	0.62	0.62	0.7	0.9	0.72	0.8	0.76	0.78	0.65	0.53	0.66	1
660	0	1.36	1.85	0.62	0.49	0.72	0.55	0.58	0.58	0.64	0.9	0.66	0.76	0.7	0.72	0.6	0.49	0.6	0.95
670	0	1.4	1.85	0.61	0.47	0.7	0.54	0.56	0.56	0.63	0.85	0.65	0.74	0.68	0.74	0.6	0.48	0.59	1
700	0	1.3	1.83	0.58	0.46	0.66	0.51	0.54	0.54	0.6	0.83	0.62	0.72	0.64	0.66	0.55	0.46	0.55	0.9

**Table A5:** OD per sample in a 96 h interval for the samples containing *Chromohalobacter canadensis*.

λ, nm	k1	k24	k48	1.1	1.2	1.3	1.4	3.1	3.2	3.3	3.4	5.1	5.2	5.3	5.4	7.1	7.2	7.3	7.4
500	0	0.69	1	0.92	0.88	1	0.65	0.61	0.9	0.7	1	1.1	1	0.67	1	0.98	0.9	1	0.89
520	0	0.65	1	0.88	0.85	0.97	0.6	0.6	0.87	0.65	0.94	1	1	0.62	0.96	0.95	0.87	0.95	0.85
600	0	0.54	0.85	0.77	0.77	0.85	0.5	0.5	0.78	0.55	0.88	0.92	0.9	0.52	0.82	0.84	0.8	0.82	0.72
620	0	0.5	0.8	0.75	0.74	0.83	0.48	0.48	0.75	0.51	0.76	0.86	0.88	0.56	0.78	0.8	0.75	0.77	0.66
660	0	0.42	0.68	0.68	0.68	0.74	0.41	0.43	0.69	0.44	0.66	0.75	0.8	0.58	0.66	0.72	0.67	0.66	0.56
670	0	0.42	0.66	0.66	0.67	0.72	0.4	0.42	0.68	0.43	0.64	0.73	0.8	0.56	0.65	7	0.66	0.65	0.55
700	0	0.38	0.61	0.63	0.64	0.64	0.37	0.39	0.66	0.41	0.61	0.69	0.76	0.48	0.6	0.67	0.63	0.6	0.54

## Appendix B

The Appendix contains the experimental optical density data for the *Streptococcus mutans* samples.

**Table A6:** Probe index of the samples containing *Streptococcus mutans*.

Probe Number	Decriptor
k1	culture medium
k24	Input 24 h incubation

**Table A7:** Probe discharge conditions for the samples containing *Streptococcus mutans*.

Probe Index		Treatment Time, s	MW Power, W	Ar Gas Flow, L/min
k24		0	0	0
1.1	1.2	60	10	5
3.1	3.2	180	10	5
5.1	5.2	300	10	5
7.1	7.2	420	10	5

**Table A8:** OD per sample in 0 h interval of the samples containing *Streptococcus mutans*.

λ, nm	k1	k24	1.1	1.2	3.1	3.2	5.1	5.2	7.1	7.2
500	0	1.1	1.08	1.08	1.06	1.06	1.06	1.06	1.06	1.06
520	0	1.1	1.08	1.08	1.06	1.06	1.06	1.06	1.06	1.06
600	0	1	0.99	0.99	0.97	0.97	0.97	0.97	0.95	0.95
620	0	0.97	0.96	0.96	0.94	0.94	0.94	0.94	0.96	0.96
660	0	0.92	0.92	0.92	0.88	0.88	0.88	0.88	0.9	0.9
670	0	0.9	0.88	0.88	0.87	0.87	0.87	0.87	0.89	0.89
700	0	0.86	0.85	0.85	0.84	0.84	0.83	0.83	0.85	0.85

**Table A9:** OD per sample in a 3 h interval of the samples containing *Streptococcus mutans*.

λ, nm	k1	k24	1.1	1.2	3.1	3.2	5.1	5.2	7.1	7.2
500	0	1.1	0.87	0.88	0.87	0.88	0.9	0.9	0.96	0.96
520	0	1.1	0.87	0.87	0.87	0.87	0.9	0.9	0.96	0.96
600	0	1	0.78	0.78	0.77	0.77	0.82	0.82	0.86	0.86
620	0	0.97	0.75	0.75	0.75	0.75	0.8	0.8	0.84	0.84
660	0	0.92	0.7	0.7	0.7	0.7	0.74	0.74	0.78	0.78
670	0	0.9	0.68	0.68	0.68	0.68	0.72	0.72	0.77	0.77
700	0	0.86	0.65	0.66	0.64	0.64	0.7	0.7	0.73	0.73

**Table A10:** OD per sample in a 24 h interval of the samples containing *Streptococcus mutans*.

λ, nm	k1	k24	1.1	1.2	3.1	3.2	5.1	5.2	7.1	7.2
500	0	1.05	0.77	0.79	0.7	0.69	0.93	1.5	0.78	1.3
520	0	1.06	0.76	0.79	0.7	0.69	0.93	1.5	0.78	1.2
600	0	0.94	0.66	0.68	0.6	0.6	0.81	1	0.68	1.2
620	0	0.9	0.64	0.77	0.56	0.58	0.78	1	0.67	1.2
660	0	0.88	0.59	0.55	0.33	0.54	0.72	0.94	0.66	1.1
670	0	0.87	0.58	0.54	0.52	0.52	0.7	0.9	0.6	1.1
700	0	0.8	0.56	0.52	0.5	0.5	0.67	0.88	0.57	1.1
